# Third molar surgical difficulty scales: systematic review and preoperative assessment form

**DOI:** 10.4317/medoral.24951

**Published:** 2021-12-07

**Authors:** Cosme Gay-Escoda, Alba Sánchez-Torres, Jordi Borrás-Ferreres, Eduard Valmaseda-Castellón

**Affiliations:** 1 MD, DDS, MS, PhD, EBOS, OMFS. Chairman and Professor of the Oral and Maxillofacial Surgery Department, School of Medicine and Health Sciences, University of Barcelona. Director of Master’s Degree Program in Oral Surgery and Implantology (EFHRE International University / FUCSO). Coordinator and Researcher of the IDIBELL Institute. Head of Oral and Maxillofacial Surgery and Implantology Department of the Teknon Medical Centre, Barcelona, Spain; 2 DDS, MS, Master of Oral Surgery and Implantology. Associate Professor of Oral Surgery, School of Medicine and Health Sciences, University of Barcelona. Researcher at the IDIBELL Institute, Barcelona, Spain; 3 DDS. Professor of the Master’s Degree Program in Oral Surgery and Implantology, EFHRE International University/FUCSO. Postgraduate degree on Temporomandibular Disorders and Orofacial Pain, SCOE, Barcelona, Spain; 4 DDS, MS, PhD, EBOS. Professor of Oral Surgery and Director of the Master’s degree program in Oral Surgery and Implantology, School of Medicine and Health Sciences, University of Barcelona. Researcher at the IDIBELL Institute, Spain

## Abstract

**Background:**

The main objective of this systematic review was to collect the pre-existing scales for assessing the difficulty of third molar extraction. The secondary objective was to design a proposal for a preoperative evaluation protocol for the difficulty of third molar extraction.

**Material and Methods:**

Two independent researchers conducted an electronic search in Pubmed (MEDLINE), Cochrane, and Scopus databases during March 2021. Included studies evaluated the prediction of the difficulty of surgical removal of impacted upper or lower third molars using new indices/scales or pre-existing scales with or without modifications. Articles referring to coronectomies or assessing pre-surgical difficulty using other tools were excluded. Neither language nor publication date restrictions were applied.

**Results:**

Out of 242 articles, 13 prospective cohort studies were finally selected. Seven developed new indices/scales, and 6 assessed the predictive ability of some pre-existing scales. Most of the indices/scales contained radiological variables and few added any patient-related variables. We proposed a preoperative assessment protocol of the difficulty of third molar extraction to facilitate treatment planning and/or considerate referral in cases of high difficulty. This proposal used patient-related, radiological and surgical variables.

**Conclusions:**

Using a preoperative protocol to evaluate the surgical difficulty, including different patient-specific, radiological and surgical variables, could facilitate treatment planning, help clinicians prevent complications and assess the possibility of referral.

** Key words:**Wisdom teeth, patient characteristics, radiological variables, surgeon experience, assessment form.

## Introduction

Removal of third molars (3M) is one of the most common procedures in oral surgery. Pre-operative evaluation of surgical difficulty can help the practitioner plan the surgical technique, estimate the operating time and foresee possible complications (1). In addition, the practitioner can also evaluate the ability to perform the surgery or, if more appropriate, refer the patient to a more qualified oral surgery specialist (2). Renton *et al*. (3) underlined the relevance of preoperative assessment of the surgical difficulty of 3Ms from a teaching point of view, since dental or radiological factors are usually more considered in preoperative training, though expert surgeons usually assess other clinical or demographic variables.

The ability to predict surgical difficulty based on the surgeon's experience is controversial, as in the published literature some studies find no difference (4), while others have even observed a trend towards better estimation of difficulty for each year of training and high values for experienced maxillofacial surgeons (5).

The fact that most 3M difficulty scales are mainly based on radiological criteria constitutes a gap between the impact that patient or surgeon factors can have on actual surgical difficulty (6). In this regard, the American Association of Endodontists has developed an assessment form called ‘Endodontic Case Difficulty Assessment Form and Guidelines’ to be used in endodontic curricula as a guide for teachers to assist students in making a correct decision process.

In the field of oral surgery there is no form to determine the difficulty and assess the ability to perform surgery or to refer the case to a specialist according to the different variables involved, such as patient, radiological and operative factors, as determined by a recently published systematic review (6). Considering that diagnosis of third molars is usually performed in primary care services, a tool to assess the difficulty of third molar extractions could help both general dental practitioners and more experienced surgeons select the proper setting for third molar extractions.

The main objective of this systematic review was to collect the pre-existing scales to assess the difficulty of 3M extraction. The secondary objective was to design a 3M difficulty assessment form, based on the previously demonstrated influencing factors, to assist clinicians, whether they are students, recent graduates or even oral surgery specialists, to make a correct treatment plan or to make a referral decision.

## Material and Methods

This systematic review was carried out according to Preferred Reporting Items for Systematic Reviews and Meta-Analyses (PRISMA) guidelines (7) and the review protocol was registered in PROSPERO database (number CRD42020186643).

Inclusion criteria were studies assessing the preoperative prediction of the difficulty of impacted upper or lower 3M removal using new indices/scales or pre-existing scales with or without modifications. Articles referring to coronectomies or that had only evaluated the preoperative difficulty by means of visual analogue scales or operating time were excluded. Neither language nor publication date restrictions were applied.

Two independent researchers (AST, JBF) performed an electronic search in Pubmed (MEDLINE), Cochrane, and Scopus databases during March 2021. The search strategy used was “(wisdom tooth OR third molar) AND (scale difficulty OR difficulty guideline OR difficulty form OR difficulty classification OR difficulty index)”. Articles were first selected by reading titles and abstracts, and finally, those that met the eligibility criteria were read in full text. A third researcher resolved any discrepancies (CGE). Moreover, a manual search into the references of the selected studies was also conducted to ensure that all studies related to the area of interest were collected. We calculated the degree of agreement between the researchers for article selection after the full text reading using Cohen's Kappa index.

Data was recorded in Tables to collect the following information: author and year, number of patients and third molars treated, objective of the use of a scale or index (development of a new one or evaluation of a pre-existing one), name of the index or scale, type of variables (patient, radiological or surgical) and individual items recorded by the index/scale, objective post-operative variables that help determine the difficulty, evaluator of the index/scale, surgeon(s) experience and main results. Based on this information and the factors that determine an increase in difficulty according to a previous systematic review (7), the authors designed a guide for assessing the surgical difficulty of 3M removal. The level of evidence from the included articles was scored according to the Scottish Intercollegiate Guidelines Network (SIGN) grading system (8).

## Results

**Figure 1:**
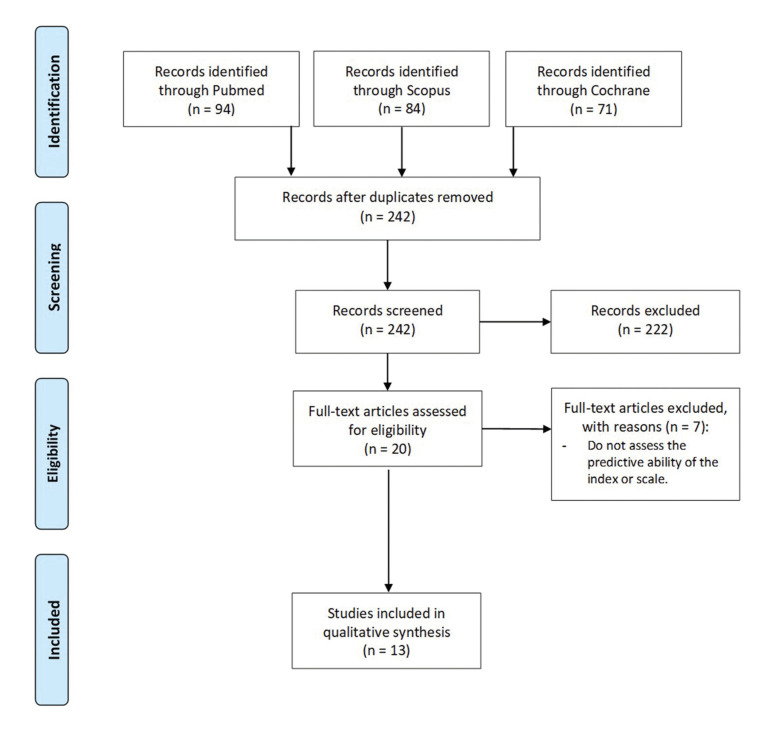
Flow-chart of the selected articles throughout the systematic review process according to PRISMA statement.

The electronic search yielded 242 articles, of which 20 were selected to be read in full text. After reading, 7 articles were excluded because they did not assess the predictive ability of the indexes/scales (9-15). Finally, 13 articles were included in the systematic review (16-28). All of them were prospective cohort studies and 1 was a multicenter study conducted in 3 centers (26). All of them assessed the difficulty of the 3M removal. Fig. 1 shows the flowchart of selected items according to PRISMA guidelines. The kappa index adjusted for bias and prevalence was 0.71, which indicated substantial agreement between researchers for article selection.

Table 1 shows the main characteristics of the included studies. Six developed new indices/scales (17,21,23-25,27) and 5 assessed the predictive ability of pre-existing indices/scales (16,18-20,22,26,28). The most widely used pre-existing index/scale, both to assess its predictability and to compare it with new indices, was Pederson scale, which includes only the radiological variables of depth, available distal space and 3M angulation. In fact, the only studies that developed indices that add variables specific to patient characteristics were those published by Roy *et al*. (21), de Carvalho and Vasconcelos (25) and Zhang *et al*. (27). Age, body mass index (BMI), mouth opening, tongue size, angle of the external oblique ridge and cheek flexibility constitute the total of patient characteristics included in these indices/scales. The rest of indices/scales evaluated included only radiological variables. Experience of the surgeon was not included in any of the indices/scales.

Most of the studies used the operative time (measured from the incision to the last suture) as a post-operative variable indicating the degree of difficulty (17,18-21,24-28). Others used scales that evaluate the type of surgical technique (16,18,20,24,25) and only 1 registered a score reported by the surgeon after the surgery to subjectively classify difficulty (23). Few studies reported on the experience of the surgeon(s) operating the cases included in the studies.

**Table 1:**
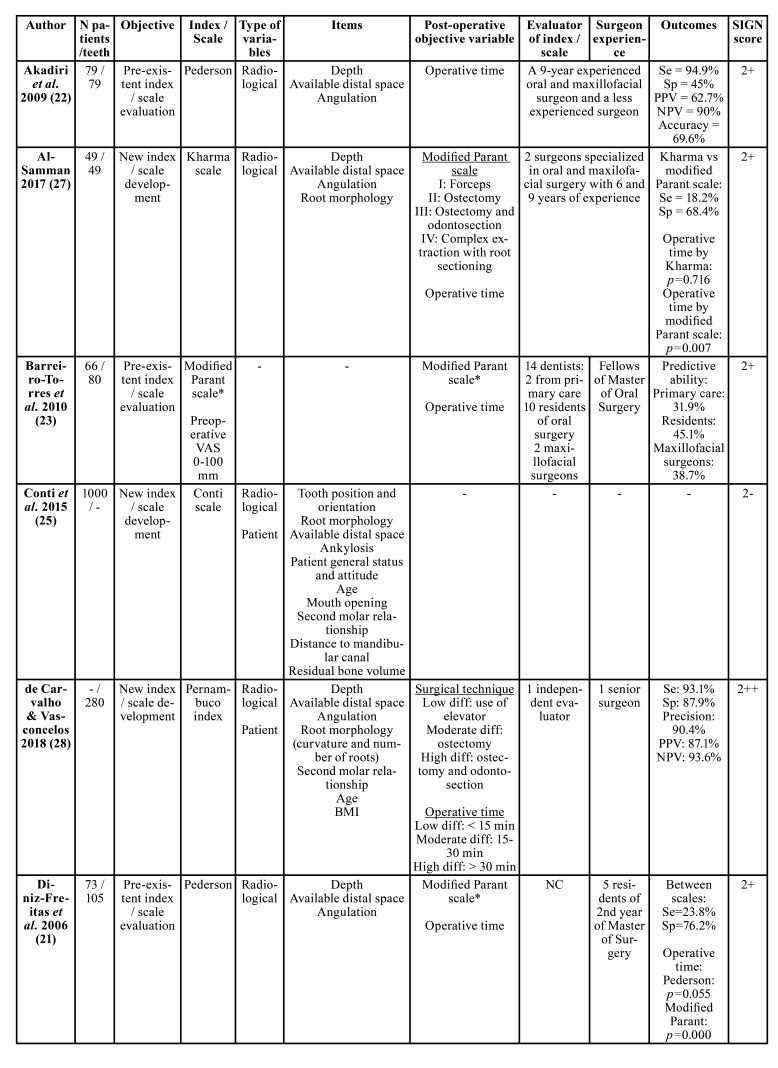
Main characteristics of the studies included.

**Table 1 cont:**
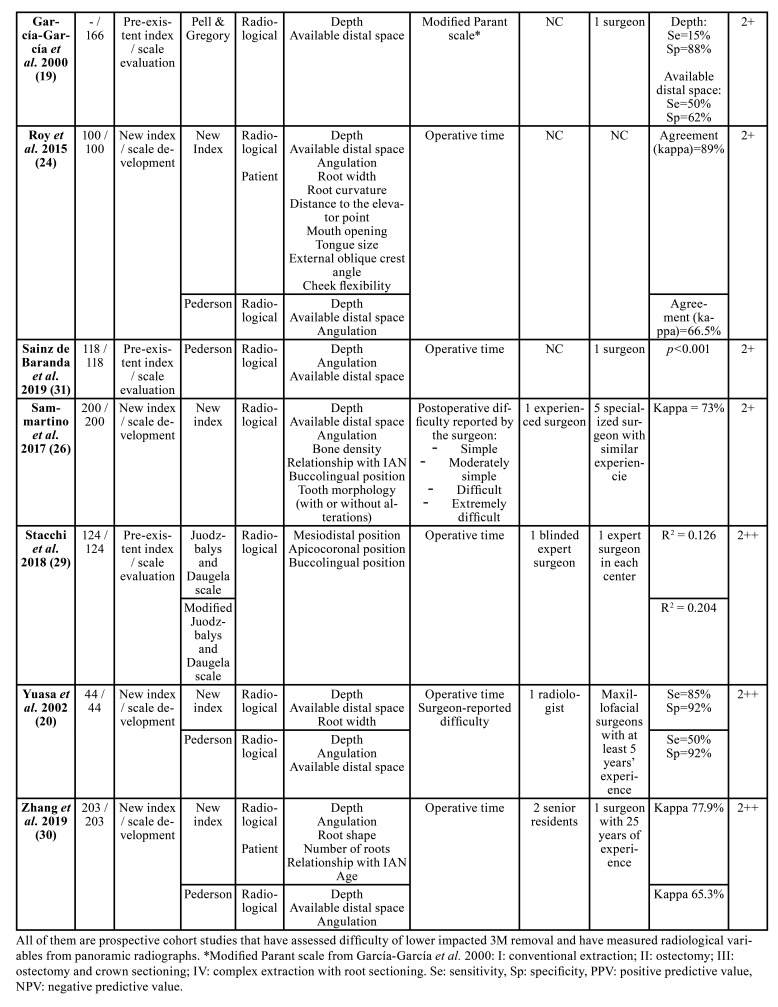
Main characteristics of the studies included.

All studies showed an improvement on the prediction of the surgical difficulty when using the new indices/scales or the proposed modifications of the pre-existing ones in comparison with pre-existing indices. Only 1 study failed to demonstrate improvement of a new index with respect to the modified Parant scale (24).

Table 2 shows the proposal of a form for the assessment of surgical difficulty of upper or lower third molars removal based on the results of the present systematic review, which combines the scales/indices developed so far, and the individual variables or factors that have been related to an increase in surgical difficulty and which have been recorded in a recently published systematic review by the authors (6). However, some of these factors have not yet been demonstrated. For this reason, the authors have completed the evaluation form with some categories based on their clinical experience in the field of oral and maxillofacial surgery.

The form includes 3 groups of variables: features of the patient, and radiological and surgical features. It classifies each clinical case into 1 out of 3 categories of difficulty. The scoring was adopted from the ‘Endodontic Case Difficulty Assessment Form and Guidelines’. Each item is scored with 1 point for low difficulty, 2 points for moderate difficulty and 5 points for high difficulty. If the sum of the points is less than 20, the case has a low difficulty, suggesting an easy surgical case, that is, a conventional extraction that can be performed by a supervised student or by a general dentist. If the sum is between 20 and 40 points, the case is classified as moderately difficult and should be operated by a dentist with training in oral surgery over 3 years or by a qualified generalist dentist with specific continuing education and over 5 years experience in oral surgery. In cases over 40 points, considered to be highly difficult, the surgical intervention should be reserved for senior surgeons with more than 10 years of experience.

**Table 2:**
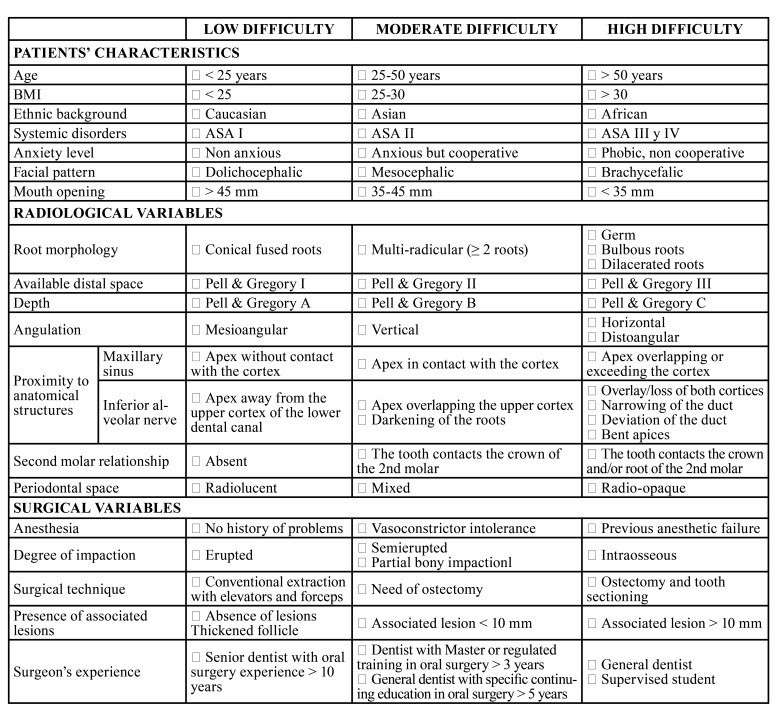
Proposed pre-surgical assessment form on surgical difficulty of upper or lower third molars removal.

## Discussion

This study aimed to collect pre-existing scales assessing the difficulty of third molars in order to design a difficulty assessment form to help professionals and to be used in an educational setting. The importance of having indices/scales that indicate the degree of surgical difficulty lies in a correct treatment planning to avoid underestimation of the difficulty and to minimize the number of intra- and post-operative complications (29). Some scales such as those of Pell and Gregory, Winter and Pederson are widely used although several studies have shown that they poorly predict surgical difficulty (16,18).

Juodzbalys and Daugela (29) carried out a literature review and designed an index/scale based on anatomical and radiological factors. This classification relates the 3M to adjacent structures such as the mandibular ramus, the second molar, the alveolar ridge, the mandibular canal, and the spatial position of the tooth. Another study published by Manuel *et al*. (30) shows a proforma for the collection of clinical history data in order to be used by residents of an oral and maxillofacial surgery service in India. The benefits of a good history are the early evaluation of difficulty and possible intra- and post-operative complications, among others. However, until now there is no specific tool to evaluate the surgical difficulty of 3M in a multidimensional way, as the one presented in this paper.

The results obtained in the present systematic review show that most of the existing indices/scales include radiological variables collected from panoramic radiographs, and only some contain variables or characteristics of the patient himself, such as age or BMI, among others. However, taking into account the results of a recently published systematic review (6), these scales are not aligned with the factors that have been shown to influence the increase in surgical difficulty. These are divided into three blocks: patient characteristics, radiological factors and surgical factors.

Surgical factors are usually treated separately from other factors. There are indices/scales that assess difficulty only by the type of surgical technique, such as the modified Parant scale (31). However, none of these refers to the surgeon's experience. The measurement of experience is a controversial issue. There are studies that refer to the number of years worked after completion of training (32) but some of those included in this review cite the senior category without explaining the number of years of experience (23,25,26). In this line, a study published by Ashton-James *et al*. (33) determines experience in terms of the number of 3M extractions performed throughout the professional career. Although few, some studies have linked the surgeon's experience with post-operative complications and morbidity and have found more complications in less experienced professionals (34,35) or non-specialized generalists (35,36), as well as greater post-operative morbidity when the procedure is performed by generalists (35).

In the field of oral and maxillofacial surgery there are no studies to assess the learning curve of the extraction of impacted 3M. The learning curve is the time and/or number of surgical interventions that a novice surgeon needs to be able to perform a procedure independently and with a good result, thus becoming a competent surgeon (37). Therefore, during this training period until the surgeon acquires the necessary skills, the risk of morbidity and complications is higher (38).

However, although the more experienced surgeons have fewer post-operative complications, the less experienced ones may also have a low number of complications, provided that their learning curve is good and progressive (39). In the area of endoscopic surgery, Qu *et al*. (40) studied the performance of surgery for an endoscopic thyroidectomy with an intra-oral approach and concluded that this competence was acquired after 20 cases, when a significant reduction in operative time was observed. In their study they detail some of the more challenging surgical steps and therefore recommend that a novice surgeon initially imitate and practice under the close guidance of an experienced supervisor. Unfortunately, the number of interventions required to master or be competent at extracting 3M is unknown as this issue has not been studied in our field. In addition, it should be noted that individual learning will depend on the surgeon's own manual skill and knowledge of anatomy or technique. Usually, as the clinician acquires skills, the difficulty of the cases increases, which can have a temporary negative impact both in complication rates and operative time (37).

In our opinion, the learning curve on difficulty assessment has to be developed also during the first years of clinical practice, both for generalists and for specialists in oral and maxillofacial surgery. Correctly predicting the difficulty of the impacted 3M removal is relevant in order to avoid iatrogeny in less expert surgeons and perform a progressive learning curve.

Therefore, the development of the present form for assessing the difficulty of surgical extraction of 3Ms based on the available scientific evidence and the clinical experience of the authors is an opportunity to improve the training of students and to guide recent graduates and even oral surgery specialists. It is intended to help reduce intra- and post-operative complications and to assist with referral to an experienced surgeon.

## Conclusions

The existing indices/scales are mainly based on radiological variables that can be evaluated in a panoramic radiography. Very few authors introduce variables related to the patient's own clinical characteristics. The few scales that evaluate surgical variables only include the type of surgical technique. None of them values the surgeon's experience.

The use of a protocol designed to evaluate the difficulty of 3Ms removal that includes patient-specific, radiological and surgical variables can facilitate treatment planning, help the professional foresee possible complications and decide whether to refer the patient to a specialist with proven knowledge and experience.
